# Effects of surgeon specialization on the outcome of emergency colorectal surgery

**DOI:** 10.1097/MS9.0000000000002685

**Published:** 2024-11-13

**Authors:** Nahar A. Alselaim, Ohood H. AlAamer, Mohammed M. Almalki, Abdualziz A. Al-osail, Sultanah F. Bin Gheshayan

**Affiliations:** College of Medicine, King Saud bin AbdulAziz university for Health Sciences, Riyadh, Saudi Arabia. King Abdullah International Medical Research Center, Riyadh, Saudi Arabia. Ministry of National Guard - Health Affairs, Riyadh, Saudi Arabia

**Keywords:** colorectal, emergency, surgeon

## Abstract

**Background:**

Colonic emergencies remain a major life-threatening condition associated with high morbidity and mortality rates. Unlike elective colorectal surgical procedures, a large portion of emergency colorectal surgical procedures are performed by noncolorectal surgeons (NCRS). The impact of specialization on the outcome of emergency colorectal surgery has not yet been well described. The authors aimed to evaluate the impact of surgeon specialization on the outcomes of emergency colorectal surgeries.

**Materials and methods:**

A retrospective cohort study conducted in a tertiary care center in Riyadh, Saudi Arabia between July 2008 to July 2020. Patients underwent emergency colorectal surgeries and met study inclusion criteria were identified and grouped according to the specialty of the primary surgeon: colorectal surgeons [CRS] or NCRS. Relevant study data was obtained from patient medical files. Bivariate and multivariate regression analyses were used to assess the association between the surgeons’ specialty and outcomes.

**Results:**

Of 219 included patients, there were 126 men [57.5%] and 93 women [42.4%]. Of all population 128 patients [58%] were operated on by CRS while 91 patients [42%] were operated on by NCRS. Most common procedure performed by CRS was left hemicolectomy [*n*=45, 67.2%] while the most common procedure performed by NCRS was right hemicolectomy [*n*=26, 51%]. The most common reason for surgery was malignant pathologies [*n*=129, 58.9%]. Patients who had their surgeries performed by a CRS had a significant decrease in 30-day mortality [odds ratio [OR] 0.23, 95% CI: 0.065–0.834]. Reoperation also decreased in this group [OR 0.413, 95% CI: 0.179–0.956]. Moreover, both hospital length of stay and ICU length of stay decreased CRS compared with the NCRS [OR 0.636, 95% CI: 0.465–0.869, and OR 0.385, 95% CI: 0.235–0.63, respectively].

**Conclusion:**

Specialization in colorectal surgery has a significant influence on morbidity and mortality after emergency operations. These findings may in improving emergency services and support remodeling the referral system in the institutions.

## Introduction

Highlights

- Malignant pathology are the most common reason for emergency colorectal surgeries.
- Specialization in colorectal surgery resulted in a decrease chance of reoperation, operative-related complications, as well as mortality rate.
- Hospital and ICU length of stay was significantly lower among patients operated by colorectal surgeons.

Colonic emergency surgeries [CES] represents a substantial proportion of emergent surgical procedures encountered in general surgery with high reported perioperative morbidity and mortality rates reported^1,2^. As colorectal specialty has a wide spectrum of patient presenting pathology from benign to malignant. Several reports indicated the factors with a high impact on perioperative morbidity and mortality outcomes. That includes major domains like patient comorbidities, advanced disease pathology, surgeon specialty, as well as the surgery burden itself^3–6^.

Considering hospitals systems and patient volume loads, subspecialized surgical units may not be available, and all different surgical cases are managed by general surgeon. In colorectal surgeries, the need for surgeon specialization has been long discussed, with most reports favoring specialized surgeon outcomes^3^. Furthermore, surgeon specialization has been linked to a better perioperative morbidity and mortality results^4,7,8^. However, surgeon-related factors and outcomes have primarily been studied in the elective based surgery of colorectal cancer, revealing differences in terms of curative resection rates, local recurrence, and survival that reflect the degree of specialization^9–11^. In 2013, the National Emergency Laparotomy Audit [NELA] was established in Great Britain to enhance the quality of care for patients undergoing emergency laparotomies^9^. According to NELA, surgeons with special interests performing emergency general surgeries out of the scope of their specialties is a potential cause of increased rates of morbidity and mortality. Therefore, the relation between surgeon subspecialty and morbidity/mortality rates following emergency colorectal surgeries warrants investigation^12^.

Globally, in many established centers, the emergency colorectal surgical cases are managed by general surgeon regardless of specialty interest. We hypothesis that the surgeon specialization has a great impact on the outcomes of the emergency colorectal cases. This study aimed to determine the impact of a surgeon’s special interest on morbidity and mortality outcomes following emergency colorectal surgeries.

## Methods

This is retrospective cohort study was conducted at a tertiary health center in Riyadh, Saudi Arabia. The study included all patients from July 2008 to July 2020 who were older than 18 years of age and underwent an emergent colorectal surgery. The procedures encompassed right hemicolectomy, left hemicolectomy [including anterior resection and sigmoid colectomy], subtotal or pan-proctocolectomy, Hartmann’s procedure and stoma creation or stoma revision. Patients who underwent concurrent surgery from another specialty were excluded, to purely examine the colorectal procedure outcome. The included patients were divided according to the surgeon’s subspecialty [CRS versus NCRS]. All data were extracted from electronic medical records.

Following institutional review board approval, applying STROCSS guidelines, patients files were utilized to collect the data for all emergency colorectal surgeries. Data were categorized into preoperative, intraoperative, and postoperative variables. Preoperative variables encompassed diagnosis, demographics [age, sex, and BMI], white blood cell count, presence of sepsis or septic shock, advanced ASA class of 3 or more, smoking status, anticoagulation use, steroid use and patient comorbidities such as pulmonary [dyspnea, chronic obstructive pulmonary disease, and mechanical ventilation requirement], cardiac [history of hypertension or congestive heart failure], hepatic [presence of ascites], renal [renal failure and dialysis requirement], and diabetes mellitus. Intraoperative variables included the amount of blood loss, stoma creation, and the type of colorectal intervention [open versus laparoscopic] and resection [total versus segmental resection]. Postoperative variables included length of stay, surgical site infection, 30-day mortality, postoperative morbidity, readmission, reoperation [return to the theater], and the length of ICU stay. Data analysis was performed using the Statistical Package for Social Sciences, version 23.0 [IBM Corp.] software.

Frequencies and percentages were utilized to display categorical variables, mean, and SD were used to display continuous variables. The *χ* ^2^ test was used to test for the presence of an association between categorical variables. Bivariate and multivariate logistic regression were utilized to determine factors predicting 30 days-mortality, reoperation, hospital length of stay, readmission, and complications. Similarly, bivariate and multivariate Poisson regression was also performed to determine factors predicting hospital length of stay and ICU length of stay. The predicting factor in the bivariate analysis was the surgeon’s specialty. The models of multivariate regression included the following factors [sex, age, BMI, diagnosis, ASA class, urgency, preoperative sepsis, surgeon specialty, procedure, stoma, and approach] to adjust the predictability of surgeon specialty in the presence of confounders. The level of significance was set at 0.05.

## Results

A total of 219 patients who met the inclusion criteria (age of 15 years or more with no other concomitant surgery) were included in the study. Of these, 128 patients [58%] were operated on by CRS while 91 patients [42%] were operated on by NCRS. Baseline characteristics of both patient groups are recorded as mentioned in Table 1. No significant statistical differences were found between the two groups regarding age, sex, BMI, comorbidities, and smoking. However, a significant difference was observed in the diagnosis of patients; a higher rate of malignant conditions was operated on by CRS compared to NCRS [69.8% vs. 30.2%, *P*<0.001]. Additionally, preoperative sepsis was significantly higher in patients operated on by NCRS compared to those operated on by CRS [39.6% vs. 20.3%, *P*=0.002].

**Table 1:** The association between patients’ characteristics and operating surgeon’s specialty

Patients characteristics	Surgeon specialty		*P* *
	Colorectal surgeon [*n*=128]	Noncolorectal surgeon [*n*=91]	
Age			0.454
18–49 years	32 [25.0%]	25 [27.4%]	
50–64 years	46 [35.9%]	24 [26.4%]	
65–74 years	22 [17.2%]	21 [23.1%]	
75 and older	28 [21.9%]	21 [23.1%]	
Sex			0.648
Male	72 [56.3%]	54 [59.3%]	
Female	56 [43.8%]	37 [40.7%]	
BMI			0.157
Underweight [BMI <18.5]	0 [0%]	3 [3.3%]	
Normal weight [BMI=18.5–24.9]	50 [39.1%]	37 [4.7%]	
Overweight [BMI=25–29.9]	44 [34.4%]	21 [23.1%]	
Obesity class 1 [BMI=30–34.9]	23 [18%]	21 [23.1%]	
Obesity class 2 [BMI=35–39.9]	8 [6.3%]	5 [5.5%]	
Obesity class 3 [BMI=40 and higher]	3 [2.3%]	4 [4.4%]	
Smoking	15 [11.7%]	11 [12.1%]	0.934
Pulmonary comorbidity	24 [54.5%]	20 [45.5%]	0.557
Cardiac comorbidity	74 [56.9%]	56 [43.1]	0.580
Hepatic comorbidity	9 [50%]	9 [50%]	0.448
Renal comorbidity	20 [52.6%]	18 [47.4%]	0.424
Endocrine comorbidity	60 [53.6%]	52 [46.4%]	0.134
Hematology comorbidity	15 [62.5%]	9 [37.5%]	0.669
Neurology comorbidity	18 [62.1%]	11 [37.9%]	0.671
Diagnosis			<0.001
Nonmalignant	38 [42.2%]	52 [57.8%]	
Malignancy	90 [69.8%]	39 [30.2%]	
Preoperative sepsis	26 [20.3%]	36 [39.6%]	0.002
WBC count			0.096
Leukopenia	7 [5.5%]	4 [4.4%]	
Normal WC count	88 [68.8%]	51 [56.0%]	
Leukocytosis	33 [25.7%]	36 [39.6%]	
ASA class			0.108
ASA class 1 and 2	50 [65.8%]	26 [34.2%]	
ASA class 3, 4, and 5	78 [54.5%]	65 [45.5%]	

* Significant at level 0.05.

Regarding surgical interventions, NCRS are more likely to opt for surgery within 48 h of presentation as compared to CRS, who allow a longer observation period [84.6% vs. 77.7%, *P*=0.182], respectively (this could be explained by patient presentation above). Moreover, NCRS are more inclined to use an open technique rather than a laparoscopic approach [85.7% vs. 77.3%, *P*=0.121]. The most common procedure performed by CRS was a left hemicolectomy [*n*=45, 67.2%], while a right hemicolectomy was predominant among NCRS [*n*=26, 51%]. CRS also had a higher tendency for stoma formation more than NCRS [80.5% vs. 76.9%, *P=*0.526]. However, no statistically significant differences were found between the two groups regarding the type of procedure type (Table 2).

**Table 2:** Surgical interventions

Surgical intervention	Surgeon specialty		*P* *
	Colorectal surgeon [*n*=128]	Noncolorectal surgeon [*n*=91]	
Timing of surgery			
<48 h	99 [77.3%]	77 [84.6%]	0.182
>48 h	29 [22.7%]	14 [15.4%]	
Type of intervention			
Open	99 [77.3%]	78 [85.7%]	0.121
Laparoscopic	29 [22.7%]	13 [14.3%]	
Performed procedure			
Right hemicolectomy	25 [49%]	26 [51%]	0.187
Left hemicolectomy	45 [67.2%]	22 [32.8%]	
Subtotal and total proctocolectomy	8 [61.5%]	5 [38.5%]	
Hartmann’s procedure	21 [65.6%]	11 [34.4%]	
Stoma formation	27 [55.1%]	22 [44.9%]	
Others	2 [28.6%]	5 [71.4%]	
Stoma creation	103 [80.5%]	70 [76.9%]	0.526

* Significant at level 0.05

Postoperative outcomes are presented in Table 3. Significant statistical differences were observed between the two groups. Patients in the CRS group experienced shorter hospital stays [11 days vs. 16 days *P*=0.001] and ICU stays [2 days vs. 8 days, *P*=0.001] compared to the NCRS group. Additionally, they had a lower rate of SSI [28.6% vs. 11.7%, *P*=0.002], reoperation [13.3% vs. 27.5%, *P*=0.009], and 30 days mortality [20.9% vs. 5.5%, *P*=0.001]. However, no significant differences in readmission or complication rates were found between the two groups.

**Table 3:** The association between surgeon specialty with postoperative outcome

Variable	Colorectal [*n*=128]	Noncolorectal [*n*=91]	*P* *
Hospital length of stay [in days] median, 25th precentile,75th percentile]	11 [7–17.75]	16 [8–37]	0.001
ICU length of Stay [median, 25th precentile,75th percentile]	2 [2–6]	8 [3–20.75]	0.001
SSI	15 [11.7%]	26 [28.6%]	0.002
30 days mortality	7 [5.5%]	19 [20.9%]	0.001
Readmission	11 [8.6%]	8 [8.8%]	0.959
Reoperation	17 [13.3%]	25 [27.5%]	0.009
Complication	29 [22.7%]	31 [34.1%]	0.062
Postoperative mortality	7 [5.5%]	19 [20.9%]	0.001

* Significant at level 0.05

A multivariable regression analysis was used to control for potential confounding factors like patient factors, nature of the disease, with results presented in Table 4. Patients operated on by CRS had significantly lower odds of 30-day mortality (odds ratio [OR]=0.232, 95% CI: 0.065–0.834), reoperation (OR=0.413, 95% CI: 0.179–0.956), and shorter hospital (OR=0.636, 95% CI: 0.465–0.869), and ICU lengths of stay (OR=0.385, 95% CI: 0.235–0.63). However, readmission and complications remained nonsignificant.

**Table 4:** Bivariate and multivariate logistic regression/poison regression [prediction of 30 days-mortality, reoperation, hospital length of stay, readmission, and incidence of complication based on surgeon specialty]

Demographic variable [colorectal surgeon vs noncolorectal surgeon]	*P* *	Unadjusted OR/IRR [CI]	*P* *	Adjusted OR/IRR [CI]
30 days mortality	0.001	0.219 [0.088–0.547]	0.025	0.232 [0.065–0.834]
Reoperation	0.01	0.404 [0.203–0.804]	0.039	0.413 [0.179–0.956]
Hospital length of stay	<0.001	0.531 [0.385–0.733]	0.004	0.636 [0.465–0.869]
ICU length of stay	<0.001	0.342 [0.192–0.608]	<0.001	0.385 [0.235–0.63]
Readmission	0.959	0.975 [0.376–2.53]	0.776	0.851 [0.279–2.596]
Complication	0.063	0.567 [0.311–1.032]	0.289	0.682 [0.337–1.383]

* Significant at level 0.05

IRR, incidence rate ratio; OR, odds ratio.

Table 5 presents the multivariate logistic regression analysis for predicting 30-day mortality. The model tested the predictability of 30-day mortality using variables such as sex, age, BMI, diagnosis, ASA class, urgency, preopeartive sepsis, surgeon specialty, procedure, approach, and stoma. The presence of characteristics such as being 50 years or older, preopeartive sepsis, and having a stoma was significantly associated with an increased risk of 30-day mortality. Conversely, undergoing an operation performed by a CRS and having a Hartmann procedure were significantly associated with a decreased risk of 30-day mortality.

**Table 5:** Multivariate logistic regression for the occurrence of 30 days mortality

Factor	*P* *	Adjusted odds ratio	CI	
Sex [Male vs. Female]	0.204	2.31	0.635	8.401
Age [18–49 is the referent]				
50–64	0.034	10.883	1.204	98.409
65–74	0.041	12.739	1.112	145.997
75 and older	0.043	10.558	1.079	103.26
BMI [normal weight is the referent]				
Overweight	0.668	1.351	0.341	5.355
Obesity 1	0.401	1.96	0.407	9.435
Obesity 2	0.09	0.081	0.004	1.473
Obesity 3	0.164	5.375	0.502	57.502
Underweight	0.999	0	0	—
Diagnosis [nonmalignant vs. malignant]	0.297	1.996	0.544	7.322
ASA class [class 1 & 2 vs. class 3, 4, and 5]	0.256	0.342	0.054	2.179
Urgency [<48 h vs. >48 h]	0.419	2.257	0.314	16.242
Preopeartive sepsis [Yes vs. No]	0.005	9.708	1.974	47.735
Surgeon specialty [colorectal surgeon vs. noncolorectal surgeon]	0.025	0.232	0.065	0.834
Procedure [right hemicolectomy is the referent]				
Left Hemicolectomy	0.066	0.162	0.023	1.129
Subtotal and total proctocolectomy	0.234	3.541	0.442	28.388
Hartmann’s procedure	0.045	0.087	0.008	0.944
Stoma formation	0.59	0.581	0.081	4.181
Others	0.34	0.222	0.01	4.888
Approach [open vs. laparoscopic]	0.351	2.99	0.299	29.873
Stoma [yes vs. no]	0.024	19.504	1.466	259.542

* Significant at level 0.05

## Discussion

The aim of this study was to examine the effect of colorectal specialization on the outcomes of emergency colorectal surgery. Our findings demonstrated that patients operated on by CRS had lower 30-day mortality, decreased hospital and ICU lengths of stay, reduced risk of reoperation, and fewer SSIs compared with patients operated on by surgeons without colorectal specialization. However, there were no significant statistical differences between the two groups regarding postoperative complications and readmission rates.

Over the past two decades, there has been a shift toward specialization within surgical specialties, particularly in elective settings. Numerous studies across various subspecialties have demonstrated improved outcomes associated with subspecialization, and colorectal surgery is no exception. Callahan *et al*.^13^ compared the outcomes of colectomies performed by subspecialty surgeons with those performed by general surgeons and found that morbidity was significantly higher in patients operated on by general surgeons. Another study, which analyzed 115 540 elective colorectal resection procedures, concluded that specialized surgeons had a lower mortality rate and a reduced length of hospital stay^14^.

Several studies have examined the impact of colorectal subspecialization on the outcomes of emergency colorectal surgery, with most demonstrating improved outcomes associated with subspecialization. A large study utilizing the NELA in England revealed that patients undergoing colorectal procedures performed by NCRS had an increased adjusted 30-day mortality risk, and the rate of return to the theater was also higher in this group^9,11^. Kulaylat *et al*. conducted a study using propensity score matching and found that cases performed by CRS were associated with significantly lower rates of 30-day mortality, postoperative morbidity, and unplanned major reoperation. Additionally, the length of stay was 4.4 days longer for patients operated on by general/acute care surgeons^7^. Boyce *et al*. focused on diverticulitis management outcomes, comparing results before and after subspecialization. They observed a decrease in operative mortality from 9.6 to 4.2% after subspecialization; the primary anastomosis rate for all left colon resections increased from 50.3 to 77.9%, and stoma formation of any type decreased from 46.6 to 27.7%^15^. Other studies have reported similar findings, with improved outcomes in patients operated on by CRS compared to those operated on by NCRS^3,8,16^.

Some studies have indicated comparable outcomes in emergency colorectal surgery, whether performed by CRS or NCRS. An Australian study of 196 consecutive patients undergoing emergency left colonic resection revealed no significant difference in 30-day mortality between the two groups. Secondary morbidity markers, such as length of stay, return to theater, anastomotic leak rate, wound issues, and systemic complications, were also similar. However, the CRS group had a significantly higher rate of primary anastomosis (85.5% vs. 28.7%) and a lower stoma rate (40.4% vs. 88.8%)^17^. Arnarson *et al*. conducted a retrospective population study using data from the Swedish Colorectal Cancer Registry, which included 656 patients undergoing emergent surgery for colon cancer. They found no significant differences in 30-day mortality, 90-day mortality, or postoperative complication rates between the CRS and NCRS groups^18^. Additionally, a Swedish study examining the impact of surgeon specialty on the outcomes of emergency colorectal cancer surgery included 235 patients and reported no difference in postoperative complications between colorectal and acute care surgeons. However, patients operated on by general surgeons had significantly higher postoperative complications, with an OR of 2.5^19^. Kulaylat *et al*.’s subgroup analysis yielded similar findings, with both general surgeons and other general surgery subspecialties experiencing significantly higher postoperative morbidity compared to CRS. In contrast, acute care and trauma surgeons had outcomes comparable to those of CRS^7^. These results suggest that frequent exposure to a set of procedures may be a more significant factor than specialization itself.

Several theories have emerged to explain the favorable outcomes of emergency colorectal surgery when performed by CRS compared to NCRS. One theory suggests that better outcomes correlate with a higher volume of specific procedures. A study from Sweden demonstrated that the adjusted OR for unplanned readmissions was six times higher for patients operated on by surgeons with the lowest resection volumes compared to those with the highest resection volumes^19^.

Another theory posits that specialty training, in itself and regardless of case volume, is associated with better outcomes in emergency colorectal surgery. Several studies have demonstrated improved outcomes with subspecialty training, independent of case volume^10,13,14,20^. A large systematic review examining the effects of both surgeon specialization and volume on patient outcomes showed that both factors are associated with improved patient outcomes^21^. Another factor suggested to influence postoperative outcomes is hospital volume. However, some studies have suggested that improved outcomes are derived from the availability of clinical services rather than from volume itself^22–24^.

This study builds on the current evidence that specialized surgeons achieve better outcomes in emergency settings, paralleling previous findings in elective settings. Moreover, there has been a shift toward specialization in elective settings over the last few decades; however, a similar transition in emergency settings is more complex. Implementing a subspecialized service in emergency colorectal surgery may be feasible in large hospitals with sufficient surgeons, but this is not the case in small or rural hospitals. In this study, we did not classify the NCRS based on their subspecialty; however, several reports have found that acute care surgeons achieve outcomes comparable to those of CRS^18,19,25^.

This study has several limitations. It is retrospective in nature, with all its inherent biases. Although we attempted to limit confounding factors with logistic regression, the study remains susceptible to selection bias. For instance, complex patient cases may have been referred to colorectal surgery due to the nature of their disease. In contrast, urgent and unstable patient cases might have been operated on more frequently by the on-call surgeon, regardless of specialty, given the time-sensitive nature of the surgical intervention. Moreover, unmeasured co-founders, like surgeon workload and experience are not counted. Additionally, the non the study was conducted in a tertiary and academic center with subspecialty-based services; therefore, the results might not be generalizable to community hospitals where a general surgeon regularly performs, and prefers elective surgery for colorectal disease.

## Conclusion

Postoperative morbidity and mortality outcomes as well as hospital and ICU stays differed according to surgeon specialization in patients undergoing ECRS. Specialization in colorectal surgery significantly impacted ECRS outcomes. Thus, the presence of a colorectal specialist surgeon will ultimately enhance patient outcomes and the hospital experience.

## Ethical approval

The study was approved by king Abdullah international medical research centre Ref.No.IRBC/1516/19.

## Consent

Not applicable.

## Source of funding

This project received no funding from funding agencies in the public, commercial, or not-for-profit sectors.

## Author contribution

O.A., M.M.A., A.A.A., and S.B.G.: data collection and manuscript preparation; N.A.: study conception, design, and manuscript preparation.

## Conflicts of interest disclosure

The authors declare no conflicts of interest.

## Research registration unique identifying number (UIN)

This paper did not involve any direct intervention with human participants, and all data was accessed through hospital records retrospectively.

## Guarantor

Ohood AlAamer.

## Data availability statement

The data that support the findings of this study are available from the corresponding author, [Ohood AlAamer], upon request.

## Provenance and peer review

Not commissioned, externally peer-reviewed.
